# Limited predictive value of bioelectrical phase angle for the development of sarcopenia in older Europeans

**DOI:** 10.1016/j.jnha.2024.100386

**Published:** 2024-10-15

**Authors:** Małgorzata Pigłowska, Andrea Corsonello, Tomasz Kostka, Regina Roller-Wirnsberger, Gerhard Wirnsberger, Johan Ärnlöv, Axel C. Carlsson, Lisanne Tap, Francesco Mattace-Raso, Francesc Formiga, Rafael Moreno-González, Robert Kob, Cornel Sieber, Pedro Gil, Sara Lainez Martinez, Ronit Ben-Romano, Itshak Melzer, Paolo Fabbietti, Fabrizia Lattanzio, Agnieszka Guligowska, on behalf of SCOPE investigators

**Affiliations:** a Department of Geriatrics, Medical University of Lodz, Poland; b Department of Pharmacy, Health and Nutritional Sciences, University of Calabria, Rende, Italy; c Center for Biostatistic and Applied Geriatric Clinical Epidemiology (IRCCS INRCA), National Institute of Health and Science on Ageing, Cosenza, Ancona, Italy; d Medical University of Graz, Department of Internal Medicine, Graz, Austria; e Medical University of Graz, Division of Nephrology, Department of Internal Medicine, Austria; f School of Health and Social Studies, Dalarna University, Falun, Sweden; g Division of Family Medicine, Department of Neurobiology, Care Sciences and Society, Karolinska Institutet, Huddinge, Sweden; h Academic Primary Health Care Centre, Region Stockholm, Stockholm, Sweden; i Department of Internal Medicine, Section of Geriatric Medicine, Erasmus MC, University Medical Center Rotterdam, The Netherlands; j Geriatric Unit, Internal Medicine Department, Bellvitge University Hospital – IDIBELL - L'Hospitalet de Llobregat, Barcelona, Spain; k Department of Internal Medicine-Geriatrics, Institute for Biomedicine of Aging (IBA), Friedrich-Alexander-Universität Erlangen-Nürnberg, Erlangen, Germany; l Department of Geriatric Medicine, Hospital Clinico San Carlos, Madrid, Spain; m Recanati School for Community Health Professions at the Faculty of Health Sciences, Ben-Gurion University of the Negev, Beer-sheva, Israel; n Italian National Research Center on Aging (IRCCS INRCA), Fermo and Cosenza, Ancona, Italy

**Keywords:** Aging, Elderly, Bioimpedance analysis, Phase angle, Sarcopenia

## Abstract

**Background:**

Despite the emerging interest in phase angle (PhA), a non-invasive marker of cell hydration and nutritional status, no previous study has reported the prospective association between PhA and incident sarcopenia. Therefore, the aim of our study was to evaluate the association of baseline PhA in older subjects without sarcopenia with the development of new sarcopenia as outcome.

**Methods:**

Six-hundred ninety-six subjects without sarcopenia aged ≥75 years enrolled in an international multicenter observational study were included. Sarcopenia was assessed according to the revised EWGSOP2 criteria at baseline and in follow-up visits at 12 and 24 months. Muscle strength was assessed through the handgrip strength test using a hydraulic grip strength dynamometer, muscle mass was assessed by bioimpedance analysis (BIA) and appendicular skeletal muscle mass (ASMM) was estimated. Physical performance was assessed by Short Physical Performance Battery (SPPB).

**Results:**

Participants who developed sarcopenia were older, less educated, had higher prevalence of osteoporosis, and lower baseline cognitive function, SPPB, handgrip strength and ASMM than those without sarcopenia. Baseline PhA was significantly lower in subjects developing sarcopenia. Nevertheless, after adjusting for all potential covariates including baseline components of sarcopenia in multiple logistic regression, neither PhA as continuous variable nor different levels of PhA were any more significant predictors of sarcopenia.

**Conclusions:**

As an indicator of cells function, PhA could be a potential useful early marker in identifying older people at risk of developing sarcopenia but its practical applicability remains uncertain with the present data.

## 1 Introduction

Sarcopenia is defined as a muscle disorder characterized by progressive loss of muscle strength and muscle mass due to aging and/or chronic diseases that increases the risk of poor health outcomes such as falls, functional impairments, disability, poor quality of life, institutionalization and mortality [1]. The prevalence of sarcopenia can range from 5% to 13% in older people aged of 60–70 years and from 11% to 50% in people over 80 years [2]. Sarcopenia diagnosis is based on guidelines proposed by the European Working Group on Sarcopenia in Older People (EWGSOP) in 2010 [3] and its revised consensus was prepared in 2019 (EWGSOP2) [1]. Three criteria are used for sarcopenia diagnosis: low muscle strength, low muscle mass, and low physical performance.

Since sarcopenia remains a serious problem worldwide, its prevention has become an important issue. In the clinical setting, using simple and reliable markers connected with muscle quality seems to be helpful in detecting sarcopenia and in triggering interventions.

Bioimpedance analysis (BIA) has been recommended by EWGSOP2 as non-invasive, inexpensive, portable, simple and valuable tool to assess muscle quantity and quality [1]. However, since BIA is an indirect method and muscle mass can solely be estimated by means of predictive equations, using bioelectrical impedance vector analysis (BIVA), basing on raw bioelectrical data has gained increasing interest. These data enables assessment of the electrical properties of tissues, such as hydration and the nutritional status in the cells [4].

A key parameter obtained from BIA in assessing muscle quality is the PhA. It is an easy to collect marker which reflects cellularity, cell membrane integrity and cell functions [5]. Several studies have described the association between PhA and muscle quantity, muscle quality, muscle strength and physical function [1,6,7]. PhA can avoid errors caused by the estimation of equation, and there is no need to assume a constant state of hydration [8]. Therefore, BIVA could be regarded as an index of overall muscle quality and a valuable tool to assess the prevalence of sarcopenia [1,[8], [9], [10]].

The expected association between PhA and sarcopenia has further been studied in different settings, different health states and diseases [[11], [12], [13], [14]]. Several existing studies show that older subjects with sarcopenia had lower PhA than subjects without sarcopenia [9,10,15,16]. Some authors aimed to find the PhA cut-off points associated with sarcopenia [14,[17], [18], [19]].

Despite an increasing number of the studies investigating the relationship between PhA and sarcopenia, no previous study has examined prospectively the real value of the PhA in predicting sarcopenia. The need to perform longitudinal studies to evaluate the effects of PhA over time on physical health has been warranted [19].

Therefore, the aim of our study was to investigate the potential role of PhA in predicting sarcopenia in a large population of older adults during two-years follow-up.

## 2 Material and methods

This analysis was performed within the framework of the SCOPE study (European Grant Agreement no. 436849), a multicenter 2-year prospective cohort study involving patients aged 75 years and more attending geriatric and nephrology outpatient services in participating institutions in Austria, Germany, Israel, Italy, the Netherlands, Poland and Spain. Methods of the SCOPE study have been extensively described elsewhere [20,21]. The study protocol was approved by ethics committees at all participating institutions, and complies with the Declaration of Helsinki and Good Clinical Practice Guidelines. Briefly, exclusion criteria were: a. Age <75 years; b. End-stage renal disease (ESRD) (eGFR < 15 ml/min/1.73 m^2^) or dialysis at the time of enrolment; c. History of solid organ or bone marrow transplantation; d. Active malignancy within 24 months prior to screening or metastatic cancer; e. Life expectancy less than 6 months; f. Severe cognitive impairment (Mini Mental State Examination < 10); g. Any medical or other reason (e.g., known or suspected inability of the patient to comply with the protocol procedure) in the judgement of the investigators; h. Unwilling to provide consent and those who cannot be followed-up. Written informed consent of each patient was obtained before entering the study. All participants underwent an extensive baseline visit including routine laboratory analysis and comprehensive geriatric assessment (CGA). The baseline visit was followed by follow-up visits at 12 (FU-12) and 24 (FU-24) months with intermediate phone contacts at 6 and 18 months. In the present study, data from intermediate phone contacts were not used.

**Fig. 1:**
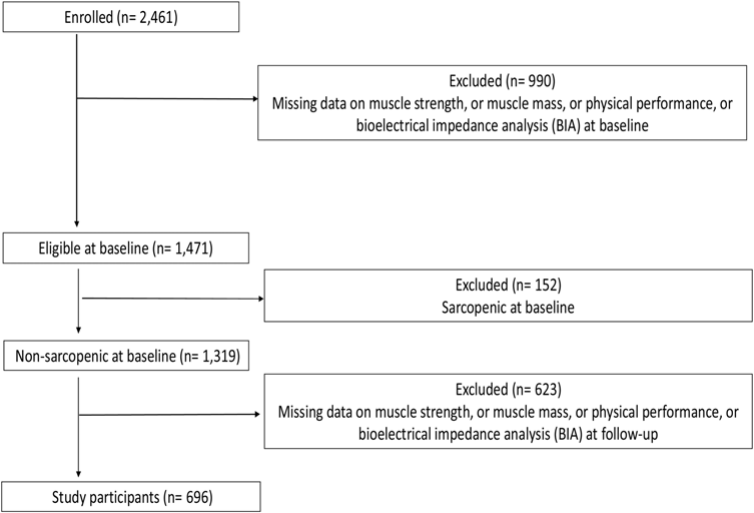
Flow-chart for study participants.

Overall, 2,461 participants were initially enrolled in the study. For the aim of the present analysis, only those participants in whom sarcopenia could be assessed in its three components (muscle strength, muscle mass and physical performance) were considered. Muscle strength assessed by handgrip strength; muscle mass by BIA; and physical performance by the Short Physical Performance Battery (SPPB) were available at baseline for 2,267 (92.1%), 1,514 (61.5%) and 2,395 (97.3%) participants, respectively. Participants with missing data were excluded from the study group. It was connected with being physically unable or unsteady, presenting arthralgia or arthritis; subjects with an implanted cardioverter-defibrillator or pacemaker were not examine due to BIA requirements. Out of 1,471 participants with complete data 152 patients were diagnosed with sarcopenia at baseline and they were excluded from further statistical analyses. Of 1,319 subjects without sarcopenia at baseline, 750 participants had complete BIA analyses at FU-12 and FU-24. Fifty-four participants had handgrip or SPPB data missing at FU-12 or FU-24 and 696 subjects were finally enrolled in the present study (Fig. 1). A comparison between included vs. excluded subjects (in two different ways) is presented in Supplementary Tables S1 and S2.

Sociodemographic and clinical characteristics were analysed for all the participants. Anthropometric measures were collected and body mass index (BMI) was calculated [22]. Several tests included in the Comprehensive Geriatric Assessment (CGA) were performed such as: Basic Activities of Daily Living (BADL) [23], Instrumental Activities of Daily Living (IADL) [24], Mini Nutritional Assessment (MNA) [25], Mini Mental State Examination (MMSE) [26], Geriatric Depression Scale (GDS) [27] and EuroQol-5D questionnaire (EQ-5D) [28].

### 2.1 Assessment of bioelectrical parameters

A whole-body composition analysis was performed using the AKERN BIA 101 New Edition 50 kHz monofrequency device (AKERN SRL, Florence, Italy). The measurement was done with the subjects in a supine position, and four adhesive skin electrodes were placed on the dorsal surfaces of hand and foot. The examination was performed on the right side of the body and on the opposite side of the body in cases of having metal surgical implants. The participants completed a minimum of eight hours of fasting before the measurement. The raw bioelectrical data: resistance (R) and reactance (Xc) were evaluated and PhA was calculated automatically by the BIA device according to the following formula: PhA (°) = −Arctang (Xc/R) × 180 × π. R and Xc were given in Ohms and PhA was given in degrees.

Assessment of sarcopenia

Sarcopenia was assessed according to the revised EWGSOP2 criteria for an operational definition of sarcopenia [1]. All three components, i.e. muscle strength, muscle mass, and physical performance were assessed. The number of sarcopenia cases was defined as the sum of confirmed sarcopenia (two criteria met) and severe sarcopenia (all three criteria met) both at baseline and follow-up.

Muscle strength was assessed through the handgrip strength test [29], using a hydraulic grip strength dynamometer (Model J00105 JAMAR Hydraulic Hand, Lafayette Instrument Company, USA). Participants were encouraged to squeeze the device as hard as they could, 3 attempts were allowed for each hand alternating sides and the maximum measurement was registered. Following the EWGSOP2 recommendations the cut-off points for low muscle strength were used, <27 kg for men and < 16 kg for women.

Muscle mass was assessed by BIA. Appendicular skeletal muscle mass (ASMM) was estimated as recommended by the EWGSOP2 consensus using the Sergi et al. equation [30], a cross-validated equation for standardisation specifically derived from older European populations:

ASMM (kg) = −3.964 + (0.227 × Ht2/R) + (0.095 × Wt) + (1.384 × sex) + (0.064 × Xc)

A decision was made to apply no adjustment for body size to ASMM measures in the algorithm, as also contemplated in the consensus. Low muscle mass was defined by an ASMM < 20.0 kg for men and <15 kg for women, as suggested by the EWGSOP2 consensus.

Physical performance was assessed by SPPB [31], which is a composite test consisting of a balance test (ability to stand for 10 s with feet close together side by side, then in semi-tandem and then in full-tandem positions), a gait speed assessment (usual time to walk 4 m), and a chair stand test (time to raise from a chair and return to the seated position 5 times without using arms). A score from 0 to 4 was assigned to each test, thus summing up to a maximum total score of 12. Following the EWGSOP2 recommendations, a total score of ≤8 was considered to indicate low physical performance.

The main outcome was the occurrence of sarcopenia according to EWGSOP2 criteria during 24-months follow-up in subjects without sarcopenia at baseline. Particular elements of sarcopenia (SPPB, handgrip strength, ASMM) were alternative outcomes.

## 3 Statistical analysis

All variables were checked for normality by the Kolmogorov–Smirnov test. Continuous and normally distributes variables were expressed as means with standard deviations and non-normally distributed variables were expressed as medians with interquartile differences. Categorical variables were expressed as numbers and percent (%). The difference between categorical variables was analysed by the chi-square test or Fisher’s Exact Test, while differences in continuous variables were detected by the Mann-Whitney test. Multiple logistic regression was used to assess the association between PhA and the development of sarcopenia during the 24-month period. PhA as continuous variable and different levels of PhA were verified for the potential association with sarcopenia at 24 months with multiple logistic regression. Several models were applied, both in all the participants and the two age-and sex-adjusted groups of 45 subjects. First, the crude model (Model 1) was adjusted for age and sex (Model 2) and for variables which were significantly different in participants with and without sarcopenia: education, MMSE and osteoporosis (Model 3). Then, the predictive value of PhA was compared with handgrip (Model A), ASMM (Model B) and SPBB (Model C) in separate models and with all three components of saropenia in one model (Model D). Finally, components of sarcopenia were added to the Model 3 separately (Models 3A, 3B, 3C) and together to construct the final model with all the potential confounders (Model 3D). The results are shown as odds ratios (OR) with the corresponding 95% confidence intervals (CI).The Receiver Operating Characteristic (ROC) curve was used to check which cut-off point of PhA that had the best combination of sensitivity and specificity to predict the occurrence of sarcopenia. The area under the ROC curve (AUC) was used as a measure of the prediction of sarcopenia. Statistical significance was set at p < 0.05. All statistical analyses were performed with SPSS version 24 (SPSS Inc., Chicago, IL, USA) and MedCalc (JMP® statistics software, USA).

## 4 Results

Forty-five older participants developed sarcopenia after 24 months of follow-up. Table 1 presents general characteristics at baseline according to occurrence of sarcopenia at 24 months (only participants without sarcopenia at the baseline assessment, with all necessary tests completed in the follow-up study were evaluated). Median age of participants who developed sarcopenia was significantly higher than of those who remained without sarcopenia (p < 0.001). Sex did not differ significantly between groups. The number of years of education were 2 years shorter in those with sarcopenia, compared to in those without sarcopenia. Participants developing sarcopenia also exhibited lower results in the MMSE test. Subjects developing sarcopenia had a higher prevalence of osteoporosis. Particular components of sarcopenia (handgrip strength, ASMM, SPPB) were significantly lower in subjects who developed sarcopenia. Out of BIA raw electrical data, R was higher and the PhA was significantly lower in participants developing sarcopenia. Higher percentages of future sarcopenic subjects were also below several baseline PhA cut-off points – between 4.0 ° and 4.7 °. At baseline, PhA was significantly associated with handgrip strength and SPPB in males, and with handgrip strength, ASMM and SPPB in females.

**Table 1:** General characteristics of the participants at baseline according to the occurrence of sarcopenia at 24-months follow-up.

Variable	No sarcopenia at 24-months follow-up	Sarcopenia at 24-months follow-up	P
	(n = 651)	(n = 45)	
Age (years)	78.0 (77.0; 81.0)	84.0 (77.5; 85.5)	<0.001
Sex (female), n (%)	362 (55.6)	31 (68.9)	0.082
Education level (years)	12.0 (9.0; 16.0)	10.0 (8.0; 14.0)	0.020
Marital status, n (%)			0.870
*Single*	41 (6.3)	4 (8.9)	
*Married or cohabiting*	345 (53.0)	23 (51.1)	
*Divorced or separated*	41 (6.3)	2 (4.4)	
*Widow*	224 (34.4)	16 (35.6)	
Economic status, n (%)			0.251
*Very good/good/sufficient*	535 (82.2)	40 (88.9)	
*Mediocre/bad*	116 (17.8)	5 (11.1)	
Living alone, n (%)	185 (28.4)	7 (15.6)	0.062
Quality of Life (EQ-5D - VAS)	80.0 (65.0; 88.0)	70.0 (50.0; 85.0)	0.062
BMI (kg/m^2^)	27.2 (24.7; 29.8)	26.2 (24.2; 28.9)	0.176
MNA at risk of malnutrition, n (%)	52 (8.0)	6 (13.3)	0.210
Dependency in 1 or more BADL, n (%)	16 (2.5)	3 (6.7)	0.094
Dependency in 1 or more IADL, n (%)	200 (30.7)	20 (44.4)	0.056
MMSE <24, n (%)	25 (3.8)	5 (11.1)	0.020
GDS score >5, n (%)	69 (10.6)	9 (20.0)	0.053
Hypertension, n (%)	454 (69.7)	33 (73.3)	0.611
Transient ischemic attack or stroke, n (%)	57 (8.8)	3 (6.7)	0.629
Cancer, n (%)	117 (18.0)	7 (15.6)	0.682
Osteoporosis, n (%)	141 (21.7)	16 (35.6)	0.031
Asthma, n (%)	47 (7.2)	2 (4.4)	0.482
Chronic Obstructive Pulmonary Disease, n (%)	65 (10.0)	2 (4.4)	0.223
Chronic heart failure, n (%)	96 (14.7)	3 (6.7)	0.133
Coronary artery disease, n (%)	73 (11.2)	1 (2.2)	0.058
Myocardial infarction, n (%)	53 (8.1)	1 (2.2)	0.151
Diabetes, n (%)	118 (18.1)	9 (20.0)	0.753
SPPB	11.0 (9.0; 12.0)	9.0 (7.0; 11.0)	<0.001
ASMM (kg)	18.7 (15.8; 22.3)	15.1 (14.1; 17.9)	<0.001
Handgrip strength (kg)	26.0 (20.0; 34.0)	18.0 (16.0; 22.0)	<0.001
Resistance (Ohms)	500.0 (452.0; 551.0)	544.0 (505.9; 580.2)	<0.001
Reactance (Ohms)	43.0 (37.0; 48.0)	43.5 (37.5; 48.5)	0.717
PhA (degrees)	4.8 (4.4; 5.3)	4.4 (4.0; 5.0)	0.004
PhA < = 4.0 °, n (%)	63 (9.7)	12 (26.7)	<0.001
PhA < = 4.1 °, n (%)	87 (13.4)	15 (33.3)	<0.001
PhA < = 4.2 °, n (%)	121 (18.6)	17 (37.8)	0.002
PhA < = 4.3 °, n (%)	150 (23.0)	20 (44.4)	0.001
PhA < = 4.4 °, n (%)	185 (28.4)	24 (53.3)	<0.001
PhA < = 4.5 °, n (%)	228 (35.0)	26 (57.8)	0.002
PhA < = 4.6 °, n (%)	263 (40.4)	27 (60.0)	0.010
PhA < = 4.7 °, n (%)	299 (45.9)	29 (64.4)	0.016

EQ-5D - VAS: Visual Analogue Scale of the EuroQol-5D questionnaire; BMI: body mass index; MNA: Mini Nutritional Assessment; BADL: Basic Activities of Daily Living; IADL: Instrumental Activities of Daily Living; MMSE: Mini Mental State Examination; GDS: Geriatric Depression Scale; SPPB: Short Physical Performance Battery; ASMM: Appendicular Skeletal Muscle Mass; PhA: phase angle.

The components of sarcopenia were significantly different in future sarcopenic subjects when assessed separately in males and females (Table 2). Males as well as females who developed sarcopenia were characterized with lower SPPB results, ASMM and handgrip strength than subjects without newly occurring sarcopenia.

**Table 2:** SPPB, ASMM and handgrip strength of males and females at baseline according to occurrence of sarcopenia at 24-months follow-up.

	Males				Females			
Variable	All (n = 303)	No sarcopenia at 24-months follow-up (n = 289)	Sarcopenia at 24-months follow-up (n = 14)	P	All (n = 393)	No sarcopenia at 24-months follow-up (n = 362)	Sarcopenia at 24-months follow-up (n = 31)	p
SPPB	11.0 (9.0; 12.0)	11.0 (9.0; 12.0)	9.0 (7.8; 11.2)	0.014	11.0 (9.0; 12.0)	11.0 (9.0; 12.0)	9.0 (7.0; 11.0)	<0.001
ASMM (kg)	22.5 (20.7; 24.3)	22.6 (20.8; 24.5)	19.7 (18.2; 21.1)	<0.001	16.0 (14.6; 17.6)	16.1 (14.7; 17.8)	14.7 (13.6; 15.2)	<0.001
Handgrip strength (kg)	34.0 (30.0; 40.0)	34.0 (30.0; 40.0)	30.0 (19.7; 30.2)	<0.001	21.0 (18.0; 24.0)	22.0 (18.9; 24.0)	16.0 (16.0; 19.0)	<0.001

SPPB: Short Physical Performance Battery; ASMM: Appendicular Skeletal Muscle Mass; kg: kilograms.

Table 3 presents the results of the multiple logistic regression showing the predictive value of PhA as continuous variable and different PhA cut-offs at baseline for the development of sarcopenia during the 24-month period. First, the crude model (Model 1) was adjusted for age and sex (Model 2). Thereafter, education, MMSE and osteoporosis, which were significantly different in participants with and without sarcopenia were added for adjustment (Model 3). PhA as continuous variable was a predictor of sarcopenia only in the crude model. Several levels of PhA were verified and three PhA cut-offs equal to 4.0 °, 4.1 ° and 4.4 ° were found to be significant predictors of sarcopenia in age-sex and fully adjusted models.

**Table 3:** Association between phase angle and phase angle cut-offs with occurrence of sarcopenia at 24-months follow-up. Logistic regression models with age, sex, education, MMSE and osteoporosis.

	Occurrence of at least 1 sarcopenia at 24-months follow-up		
Predictors	Model 1 OR (95%CI)	Model 2 OR (95%CI)	Model 3 OR (95%CI)
PhA, degrees	**0.61 (0.40 – 0.93)**	0.78 (0.53–1.14)	0.72 (0.49–1.07)
PhA, < = 4.0°	**3.39 (1.67–6.90)**	**2.31 (1.09–4.89)**	**2.45 (1.14–5.26)**
PhA, < = 4.1°	**3.24 (1.68–6.27)**	**2.27 (1.13–4.55)**	**2.38 (1.17–4.86)**
PhA, < = 4.2°	**2.66 (1.41–5.01)**	1.73 (0.87–3.41)	1.86 (0.93–3.72)
PhA, < = 4.3°	**2.67 (1.44–4.94)**	1.77 (0.91–3.42)	1.83 (0.94–3.57)
PhA, < = 4.4°	**2.88 (1.56–5.30)**	**1.99 (1.04–3.79)**	**2.10 (1.09–4.03)**
PhA, < = 4.5°	**2.54 (1.37–4.69)**	1.70 (0.88–3.26)	1.87 (0.96–3.64)
PhA, < = 4.6°	**2.21 (1.19–4.10)**	1.51 (0.79–2.90)	1.70 (0.88–3.31)
PhA, < = 4.7°	**2.13 (1.14–4.00)**	1.45 (0.75–2.82)	1.59 (0.81–3.12)

OR: odds ratio; CI: confidence interval; PhA: phase angle.

Model 1 – Crude model.

Model 2 – Age and sex adjusted model.

Model 3 – Model adjusted for age, sex, education, MMSE and osteoporosis.

Table 4 shows the predictive value of PhA compared with handgrip (Model A), ASMM (Model B) and SPBB (Model C) in separate models and with all three components of saropenia in one model (Model D). PhA as continuous variable was not the predictor of sarcopenia in any model. Several levels of PhA were found to be significant predictors of sarcopenia after including separate sarcopenia components but were not any more after taking into account all the three components of sarcopenia.

**Table 4:** Association between phase angle and phase angle cut-offs with occurrence of sarcopenia at 24-months follow-up. Logistic regression models with handgrip, ASMM, and SPPB.

	Occurrence of at least 1 sarcopenia at 24-months follow-up			
Predictors	Model A* OR (95%CI)	Model B** OR (95%CI)	Model C*** OR (95%CI)	Model D**** OR (95%CI)
PhA, degrees	0.77 (0.51–1.15)	0.70 (0.45–1.10)	0.78 (0.53–1.13)	0.88 (0.61–1.28)
PhA, < = 4.0°	**2.58 (1.23–5.41)**	**3.30 (1.58–6.90)**	**2.26 (1.06–4.83)**	2.12 (0.97–4.64)
PhA, < = 4.1°	**2.51 (1.26–4.98)**	**3.11 (1.58–6.15)**	**2.16 (1.06–4.40)**	1.99 (0.96–4.13)
PhA, < = 4.2°	**2.03 (1.05–3.92)**	**2.41 (1.25–4.62)**	1.76 (0.88–3.48)	1.50 (0.74–3.04)
PhA, < = 4.3°	**2.03 (1.07–3.84)**	**2.37 (1.26–4.46)**	1.80 (0.93–3.50)	1.54 (0.78–3.03)
PhA, < = 4.4°	**2.19 (1.17–4.11)**	**2.46 (1.31–4.59)**	**2.06 (1.08–3.93)**	1.71 (0.88–3.31)
PhA, < = 4.5°	**1.93 (1.02–3.63)**	**2.10 (1.12–3.94)**	1.83 (0.96–3.49)	1.49 (0.77–2.89)
PhA, < = 4.6°	1.69 (0.89–3.19)	1.80 (0.96–3.39)	1.57 (0.82–3.02)	1.27 (0.65–2.48)
PhA, < = 4.7°	1.48 (0.77–2.85)	1.66 (0.87–3.17)	1.53 (0.79–2.97)	1.14 (0.58–2.25)

OR: odds ratio; CI: confidence interval; PhA: phase angle.

* Adjusted for handgrip.

** Adjusted for ASMM.

*** Adjusted for SPPB.

**** Adjusted for handgrip, ASMM, and SPPB.

Finally, in the Table 5 components of sarcopenia were added to the Model 3 separately (Models 3A, 3B, 3C) and together to construct the final model with all the potential confounders (Model 3D). Neither PhA as continuous variable nor at any PhA cut-off point was the predictor of future sarcopenia in fully adjusted model.

**Table 5:** Association between phase angle and phase angle cut-offs with occurrence of sarcopenia at 24-months follow-up. Logistic regression models with handgrip, ASMM, SPPB, age, sex, education, MMSE and osteoporosis.

	Occurrence of at least 1 sarcopenia at 24-months follow-up			
Predictors	Model 3A* OR (95%CI)	Model 3B** OR (95%CI)	Model 3C*** OR (95%CI)	Model 3D**** OR (95%CI)
PhA, degrees	0.84 (0.58–1.21)	0.83 (0.53–1.30)	0.80 (0.56–1.15)	1.10 (0.72–1.69)
PhA, < = 4.0°	1.80 (0.81–3.99)	**2.46 (1.10–5.49)**	1.94 (0.87–4.34)	1.48 (0.62–3.53)
PhA, < = 4.1°	1.79 (0.86–3.75)	**2.44 (1.15–5.18)**	1.88 (0.89–3.97)	1.52 (0.67–3.43)
PhA, < = 4.2°	1.38 (0.67–2.84)	1.71 (0.83–3.52)	1.46 (0.70–3.02)	1.00 (0.45–2.21)
PhA, < = 4.3°	1.39 (0.70–2.79)	1.63 (0.81–3.27)	1.45 (0.72–2.93)	0.98 (0.46–2.11)
PhA, < = 4.4°	1.61 (0.82–3.19)	1.83 (0.93–3.60)	1.73 (0.87–3.41)	1.14 (0.54–2.40)
PhA, < = 4.5°	1.49 (0.75–2.96)	1.61 (0.81–3.22)	1.54 (0.77–3.08)	1.05 (0.49–2.24)
PhA, < = 4.6°	1.35 (0.68–2.69)	1.34 (0.67–2.69)	1.38 (0.69–2.77)	0.82 (0.38–1.75)
PhA, < = 4.7°	1.18 (0.58–2.38)	1.20 (0.59–2.43)	1.30 (0.64–2.63)	0.72 (0.33–1.56)

OR: odds ratio; CI: confidence interval; PhA: phase angle.

* Adjusted for handgrip, age, sex, education, MMSE and osteoporosis.

** Adjusted for adjusted for ASMM, age, sex, education, MMSE and osteoporosis.

*** Adjusted for SPPB, age, sex, education, MMSE and osteoporosis.

**** Adjusted for handgrip, ASMM, SPPB, age, sex, education, MMSE and osteoporosis.

We performed additional analyses and created case-control study group with 45 non-sarcopenic participants, age- and sex-matched to the group with sarcopenia (45 vs. 45 subjects). The groups were not significantly different according to the majority of variables. Differences occurred only in educational level and sarcopenia parameters including R and PhA. All the logistic models were analyzed for these groups. The logistic regression model analysis revealed similar results to those obtained in the whole study group. They are presented in Supplementary Tables S3–S6.

**Fig. 2:**
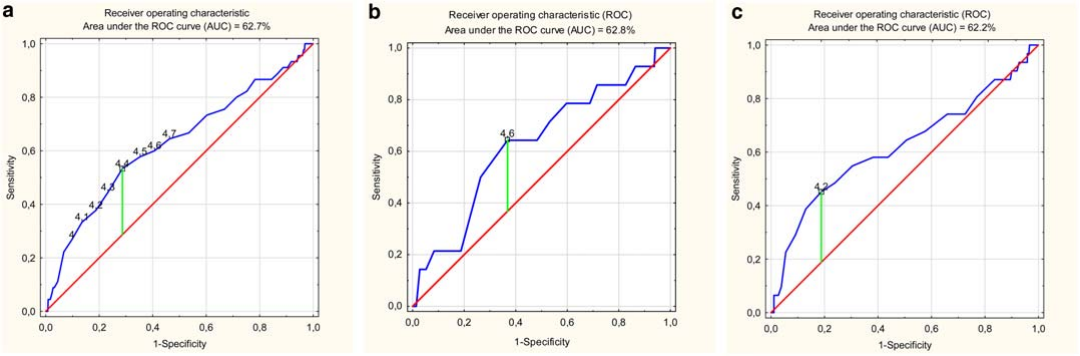
(a) ROC curve to identify the optimal phase angle cut-off for prognosing sarcopenia in the whole study group. (b) ROC curve to identify the optimal phase angle cut-off for prognosing sarcopenia in males. (c) ROC curve to identify the optimal phase angle cut-off for prognosing sarcopenia in females.

ROC analysis of the PhA score and occurrence of sarcopenia revealed a poor overall predictive value of PhA (Fig. 2a). The AUC for PhA was 62.7%. The optimal cut-off point according to ROC was 4.4 °.

Similar AUC results were obtained when ROC analyses were performed separately for men and women (Fig. 2b and 2c). The optimal cut-off point according to ROC was 4.6 ° for men and 4.2 ° for women. The AUC for PhA was 62.8% and 62.2% for men and women, respectively.

## 5 Discussion

To the best of our knowledge, this is the first prospective study investigating the role of PhA in predicting the incidence of sarcopenia. Our results suggest that low PhA level measured at baseline was related to future sarcopenia but wasn’t an independent predictor for development of sarcopenia in the two years period after adjusting for other covariates. Likewise, its overall predictive value was not strong.

Our analyses focused on a particular subset of participants, i.e. those initially identified as non-sarcopenic within the baseline group, where the prevalence of sarcopenia was 10.3%. Therefore, the 6.5% prevalence of sarcopenia in the final group was exclusively derived from new cases, representing individuals who developed sarcopenia during the 2-year follow-up period.

Patients who developed sarcopenia were older than those who did not develop sarcopenia and characterized with shorter time of education. These findings are in line with existing studies showing that the prevalence of sarcopenia increases with age and that higher age is a risk factor for sarcopenia [1,32]. Better education may influence the level of awareness of sarcopenia and a healthier lifestyle is lowering the risk of sarcopenia [33]. Cognitive function and the presence of osteoporosis were two other independent variables predicting occurrence of sarcopenia in the future. Similarly to our results, some reports found the relationship between cognitive functions and sarcopenia [34]. Osteoporosis has been described as the one of chronic diseases most often associated with sarcopenia [35].

Available research strongly supports the opinion that sarcopenia is associated with decreased PhA. The studies showed that PhA was lower in older adults with sarcopenia and that PhA was associated with sarcopenia, together with several other factors including age. These results have been obtained in various groups of participants: community-dwelling [10,[15], [16], [17], [18], [19]], hospitalized [9,14] and in different diseases, for example in patients with hepatocirrhosis [36]. Moreover, researchers revealed that PhA was not only significantly associated with sarcopenia, but also with its particular elements reflecting good accuracy in detecting patients at risk of sarcopenia [14,16,19]. The results of Japanese study [16] showed that PhA was lower in subjects with sarcopenia and that it was associated with both muscle quantity and quality. Other authors also found that PhA reflected muscle quality suggesting its utility to improve the diagnosis of sarcopenia [19]. The Italian study [14] revealed that PhA was related to muscle strength and mass both at univariate and multivariate analyses. In older people with cancer, PhA was independently connected with decreased muscle strength, impaired quality of life, and increased mortality [37]. In patients with cardiovascular disease PhA was found to be a good marker of muscle wasting and malnutrition [38].

Our results confirmed the relationship between PhA and sarcopenia in community-dwelling older adults. The prospective character of this study allowed us to be one step ahead in the analyses. The level of PhA measured at the baseline was associated with sarcopenia development. Subjects who developed sarcopenia in the 2-year period were characterized with significantly lower PhA at the beginning of the study than subjects without sarcopenia during the whole study. It suggests that initial changes ongoing in the muscles are associated with cellularity decline. Our findings also show that each component of sarcopenia (SPPB, handgrip strength, ASMM) was better at baseline in the non-sarcopenic group. Moreover, the differences were present not only in the investigated group, but also when analysed separately in males and females.

Apart from PhA, the differences were also visible in R measured with BIA at baseline. Therefore, lower PhA in participants who developed sarcopenia may indicate worse cellular integrity and cell function in this group, accompanied with increased R which is inversely proportional to body water. The potential mechanisms directly linking PhA and sarcopenia refer to the fact that skeletal muscles in sarcopenia change their composition. Decreasing the number and the size of muscle cells with integral membranes may reduce the phase shift between the voltage and the current flowing through the tissues [15,39]. The reduction of size of cells may be caused by their worse nutrition and hydration so the role of water in preserving functional status and muscle strength is important. Good cellular hydration has a protective role against weakness and functional decline, while lower myocyte hydration may lead to muscle atrophy and may be connected with lower muscle quality what is reflected in bioelectrical data including lower value of PhA [39,40]. Moreover, increased fibrosis and fat infiltration into muscles are observed in sarcopenic subjects what results in decreased strength and functionality [41,42]. Finally, the loss of membrane integrity has been described to contribute to the sarcopenic phenotype. A reduction in the lipid content of cells membranes causes membrane instability what reduces size of muscle fibers and muscle cells atrophy [15]. Thus, PhA may be a potential useful surrogate measure helping clinicians to diagnose muscle wasting.

Nevertheless, it needs to be taken into account that low results of handgrip strength, ASMM and SPPB at baseline not yet connected with sarcopenia diagnosis were also reflected by lower PhA and that all of these elements were correlated with PhA It is also worth mentioning that older patients with chronic conditions may be overhydrated what may cause errors in the muscle mass measurement used for diagnosis of sarcopenia. Furthermore, multiple logistic models revealed that baseline PhA was not independently associated with sarcopenia occurrence in subjects without sarcopenia in the two years, when adjusted for other potential co-determinants, including baseline values of distinct components of sarcopenia definition (handgrip, ASMM, SPBB). As some of these components may be easily measured (handgrip, functional tests) and they predict development of future sarcopenia there is a question of practical utility of PhA. Therefore, though PhA may be an early marker of the risk of sarcopenia in community-dwelling older adults but its applicability in a wider population use may be disputable given present data.

Several authors have aimed to find the PhA cut-off points associated with sarcopenia [[14], [15], [16], [17], [18]]. These cut-off points were different depending on the study population, age, environment or the device used. Just a few of them were performed in domestic environment. In Japanese community-dwelling older people [16] the cut-off value of the PhA for sarcopenia was 4.05 ° for men and 3.55 ° for women (medium age 81.1 years for men and 80.4 years for women) while in Japanese patients undergoing post-stroke rehabilitation PhA cutoff value for sarcopenia diagnosis was 4.76 ° in men and 4.11 ° in women (medium age 74.0 years for the whole group) [43]. Moreover, baseline PhA was associated with the recovery of functional status and dysphagia level at discharge [43]. Another study [19] showed that PhA cut-off points for sarcopenia were 5.04 for older men and 4.20 for older women living in the community (medium age 74.4 years old and 73.1 years old for men and women, respectively). In community-dwelling subjects aged 65 years and more in Mexico City, PhA cut-off for sarcopenia was ≤4.1 ° [18], while PhA cut-off value for community-dwelling and hospitalized older people in Turkey to detect sarcopenia was ≤4.55 ° [17]. Low PhA with cut-off values ≤4.95 ° for men and ≤4.35 ° for women was independently related to increased risk of incident disability in older adults [44], while in patients with rheumatoid arthritis, PhA <4.06 ° for women and <5.26 ° for men was related with falls in a 2-years prospective cohort study [45]. In our study the value of PhA in non-sarcopenic subjects was 4.8 ° in each study period (baseline, FU-12 and FU-24). In the future sarcopenic group, the baseline PhA was 4.4 ° for the whole studied group and 4.6 ° in men and 4.2 ° in women. These data seem comparable to previous studies performed either separately in men and women or in the mixed populations.

Our study demonstrated that PhA could be potentially useful biomarker in identifying older people at risk of sarcopenia development in the future but future studies should evidence its advantage against classical components of sarcopenia. Nevertheless, early detection of low PhA should raise awareness for physicians taking care of older people to perform screening tests associated with sarcopenia and to implement proper prevention to maintain muscle quantity and quality, such as education, proper diet with higher protein intake and a high physical activity level. As some researchers showed that PhA in older adults may be modulated by physical exercises [46,47], a higher physical activity level seems to be crucial.

In clinical practice, PhA may be useful as a prognostic indicator of worsening cellular integrity and number of cells. Cost-effectiveness of this measure is connected with the simplicity of obtaining PhA value in BIA measurement. As raw bioelectrical parameter, PhA is among first data displayed directly on the BIA device, with any formulas required. Thus, the process of information acquisition is non-complicated, fast and very simple. As well, its interpretation for clinicians is very simple while standardized PhA cut-off points are defined and confirmed in clinical studies for particular groups of patients. Moreover, using BIA method gives the possibility of PhA interpretation in bedridden patients – as BIA is portable and non-expensive, it is the most applicable bedside technique. Therefore, if future studies confirm its predictive value, PhA below defined cut-off points may facilitate clinical decisions and verifying effectiveness of interventions.

The main strength of our paper is the prospective design of the study and relatively large study group. Moreover, it has been focused on carefully chosen groups of participants – it has been performed in participants selected from the baseline group without sarcopenia, allowing incident cases in patients who developed sarcopenia in the 2 years follow-up.

Our study has some limitations that warrant mentioning. First, of 1,319 non-sarcopenic subjects at baseline, only 696 participants were enrolled at FU-12 and FU-24 due to missing data. It may lead to underestimation of prevalence of sarcopenia in the study. Second, ASMM has been obtained with BIA method which, despite is recommended by the EWGSOP, is not a gold standard method for the assessment of muscle mass. However, although the gold standard methods are desirable for obtaining the most accurate values of muscle mass, their high costs may lead to limited access in clinical practice and make difficult or impossible to perform effective sarcopenia screening in many patients. Therefore, in clinical practice, it is necessary to identify cost-and time-effective methods to accurately measure body composition in older patients. Since BIA is useful as a portable alternative to DXA as it is inexpensive, easy to use, and requires no radiation exposure for the patients, this method has been used in the present study. Third, ASMM used in the diagnostic criteria has not been used as adjusted for body size. Since the EWGSOP2 consensus made no recommendation to use ASMM divided by height, a decision was made to use ASMM given in kilograms. Fourth, covariates were selected based only on variables that showed significant differences in bivariate analysis. However, only 45 sarcopenia cases were found in FU-12 and FU-14 what made impossible to insert large number of variables in the model. Finally, relatively few new sarcopenia cases made difficult to set an exact cut-off point of PhA, and overall predictive value of PhA was not strong. Therefore, future prospective large studies using additional biomarkers as well as gold standard imaging methods are still required.

## 6 Conclusions

Measuring PhA as an indicator of cellular function could potentially serve as a valuable early marker in pinpointing older individuals at risk of developing sarcopenia but its practical applicability remains uncertain with the present data. Neverteless, the identification of low PhA should prompt primary care physicians, geriatricians, and other medical professionals involved in elderly care to conduct further diagnostic tests related to sarcopenia and implement appropriate preventive measures.

## Funding

The work reported in this publication was granted by the European Union Horizon 2020 program (Grant Agreement no 634869). Funding body had no role in the design of the study and collection, analysis, and interpretation of data, writing the manuscript and in the decision to publish the results.

## Ethics approval and consent to participate

The study protocol was approved by the appropriate ethics committees at all participating institutions, and have therefore been performed in accordance with the ethical standards laid down in the 1964 Declaration of Helsinki and its later amendments and Good Clinical Practice Guidelines.

## Ethics approvals have been obtained by Ethics Committees in participating institutions

Italian National Research Center on Aging (INRCA), Italy, #2015 0522 IN, January 27, 2016.

University of Lodz, Poland, #RNN/314/15/KE, November 17, 2015.

Medizinische Universität Graz, Austria, #28–314 ex 15/16, August 5, 2016.

Erasmus Medical Center Rotterdam, The Netherland, #MEC-2016–036—#NL56039.078.15, v.4, March 7, 2016.

Hospital Clínico San Carlos, Madrid, Spain, # 15/532-E_BC, September 16, 2016.

Bellvitge University Hospital Barcellona, Spain, #PR204/15, January 29, 2016.

Friedrich-Alexander University Erlangen-Nürnberg, Germany, #340_15B, January 21, 2016.

Helsinki committee in Maccabi Healthcare services, Bait Ba-lev, Bat Yam, Israel, #45/2016, July 24, 2016.

The informed consent was signed by the subject or a close relative. All methods were performed in accordance with relevant guidelines and regulations.

## Informed consent

All participants gave their informed consent prior to their inclusion in the study.

## Conflict of interests

Johan Ärnlöv has received lecturing fees from AstraZeneca and Novartis and served on advisory boards for AstraZeneca, Astella and Boehringer Ingelheim, all unrelated to the present paper. The other authors declare that they have no conflict of interests and certify that they comply with the ethical guidelines for authorship.
