# Supplementary material for: Limited predictive value of bioelectrical phase angle for the development of sarcopenia in older Europeans
Source: J Nutr Health Aging. 2024 Oct 15;28(12):100386. doi: 10.1016/j.jnha.2024.100386 (PMC12877206; doi:10.1016/j.jnha.2024.100386)
Supplement: Supplementary file 1 [file mmc1.docx]

**Supplementary Table 1.** **Comparison between included vs excluded subjects.**

|  | **Total** | | |
| --- | --- | --- | --- |
| **Variable** | **Included**  **(n =696)** | **Excluded**  **(n =623)** | **p** |
| Age (years) | 79.0 (77.0; 82.0) | 80.0 (77.0; 83.0) | <0.001 |
| Sex (female), n (%) | 393 (56.5) | 350 (56.2) | 0.917 |
| Education level (years) | 12.0 (9.0; 16.0) | 10.0 (8.0; 13.0) | <0.001 |
| Marital status, n (%) |  |  | 0.514 |
| *Single* | 45 (6.5) | 40 (6.4) |  |
| *Married or cohabiting* | 368 (52.9) | 333 (53.5) |  |
| *Divorced or separated* | 43 (6.2) | 27 (4.3) |  |
| *Widow* | 240 (34.5) | 223 (35.8) |  |
| Economic status, n (%) |  |  | <0.001 |
| *Very good/good/sufficient* | 575 (82.6) | 575 (92.3) |  |
| *Mediocre/bad* | 121 (17.4) | 48 (7.7) |  |
| Living alone, n (%) | 192 (27.6) | 124 (19.9) | 0.001 |
| Quality of Life (EQ-5D - VAS) | 79.0 (65.0; 85.0) | 70.0 (50.0; 80.0) | <0.001 |
| BMI (kg/m^2^) | 27.1 (24.7; 29.7) | 27.7 (24.8; 30.8) | 0.007 |
| MNA at risk of malnutrition, n (%) | 58 (8.3) | 86 (13.8) | 0.001 |
| Dependency in 1 or more BADL, n (%) | 19 (2.7) | 25 (4.0) | 0.195 |
| Dependency in 1 or more IADL, n (%) | 220 (31.6) | 277 (44.5) | <0.001 |
| MMSE <24, n (%) | 30 (4.3) | 53 (8.5) | 0.002 |
| GDS score >5, n (%) | 78 (11.2) | 77 (12.4) | 0.503 |
| Hypertension, n (%) | 487 (70.0) | 491 (78.8) | <0.001 |
| Transient ischemic attack or stroke, n (%) | 60 (8.6) | 62 (10.0) | 0.405 |
| Cancer, n (%) | 124 (17.8) | 118 (18.9) | 0.598 |
| Osteoporosis, n (%) | 157 (22.6) | 194 (31.1) | <0.001 |
| Asthma, n (%) | 49 (7.0) | 35 (5.6) | 0.291 |
| Chronic Obstructive Pulmonary Disease, n (%) | 67 (9.6) | 91 (14.6) | 0.005 |
| Chronic heart failure, n (%) | 99 (14.2) | 110 (17.7) | 0.088 |
| Coronary artery disease, n (%) | 74 (10.6) | 79 (12.7) | 0.246 |
| Myocardial infarction, n (%) | 54 (7.8) | 53 (8.5) | 0.619 |
| Diabetes, n (%) | 127 (18.2) | 159 (25.5) | 0.001 |
| SPPB | 11.0 (9.0; 12.0) | 9.0 (7.0; 11.0) | <0.001 |
| ASMM (kg) | 18.3 (15.6; 22.2) | 18.6 (15.6; 22.2) | 0.705 |
| Handgrip strength (kg) | 25.0 (20.0; 32.4) | 24.0 (18.0; 32.0) | 0.003 |
| Resistance (Ohms) | 505.3 (454.0; 555.3) | 489.0 (446.0; 547.0) | 0.008 |
| Reactance (Ohms) | 43.0 (37.0; 48.0) | 41.0 (34.0; 47.0) | <0.001 |
| PhA (degrees) | 4.8 (4.4; 5.2) | 4.7 (4.1; 5.2) | <0.001 |
| PhA <= 4.0°, n (%) | 75 (10.8) | 141 (22.6) | <0.001 |
| PhA <= 4.1°, n (%) | 102 (14.7) | 172 (27.6) | <0.001 |
| PhA <= 4.2°, n (%) | 138 (19.8) | 201 (32.3) | <0.001 |
| PhA <= 4.3°, n (%) | 170 (24.4) | 220 (35.3) | <0.001 |
| PhA <= 4.4°, n (%) | 209 (30.0) | 247 (39.6) | <0.001 |
| PhA <= 4.5°, n (%) | 254 (36.5) | 277 (44.5) | 0.003 |
| PhA <= 4.6°, n (%) | 290 (41.7) | 304 (48.8) | 0.009 |
| PhA <= 4.7°, n (%) | 328 (47.1) | 336 (53.9) | 0.014 |

EQ-5D - VAS: Visual Analogue Scale of the EuroQol-5D questionnaire; BMI: body mass index; MNA: Mini Nutritional Assessment; BADL: Basic Activities of Daily Living; IADL: Instrumental Activities of Daily Living; MMSE: Mini Mental State Examination; GDS: Geriatric Depression Scale; SPPB: Short Physical Performance Battery; ASMM: Appendicular Skeletal Muscle Mass; PhA: phase angle.

**Supplementary Table 2. Comparison between included vs excluded (because of unavailable follow-up data) subjects.**

|  | **Total** | | |
| --- | --- | --- | --- |
| **Variable** | **Included**  **(n =696)** | **Excluded**  **(n =165)** | **p** |
| Age (years) | 79.0 (77.0; 82.0) | 82.0 (78.0; 85.5) | <0.001 |
| Sex (female), n (%) | 393 (56.5) | 99 (60.0) | 0.409 |
| Education level (years) | 12.0 (9.0; 16.0) | 10.0 (8.0; 13.0) | <0.001 |
| Marital status, n (%) |  |  | 0.023 |
| *Single* | 45 (6.5) | 12 (7.3) |  |
| *Married or cohabiting* | 368 (52.9) | 68 (41.2) |  |
| *Divorced or separated* | 43 (6.2) | 8 (4.8) |  |
| *Widow* | 240 (34.5) | 77 (46.7) |  |
| Economic status, n (%) |  |  | 0.015 |
| *Very good/good/sufficient* | 575 (82.6) | 149 (90.3) |  |
| *Mediocre/bad* | 121 (17.4) | 16 (9.7) |  |
| Living alone, n (%) | 192 (27.6) | 41 (24.8) | 0.477 |
| Quality of Life (EQ-5D - VAS) | 79.0 (65.0; 85.0) | 70.0 (50.0; 80.0) | <0.001 |
| BMI (kg/m^2^) | 27.1 (24.7; 29.7) | 30.0 (24.5; 31.1) | 0.055 |
| MNA at risk of malnutrition, n (%) | 58 (8.3) | 30 (18.2) | <0.001 |
| Dependency in 1 or more BADL, n (%) | 19 (2.7) | 7 (4.2) | 0.307 |
| Dependency in 1 or more IADL, n (%) | 220 (31.6) | 78 (47.3) | <0.001 |
| MMSE <24, n (%) | 30 (4.3) | 15 (9.1) | 0.013 |
| GDS score >5, n (%) | 78 (11.2) | 24 (14.6) | 0.222 |
| Hypertension, n (%) | 487 (70.0) | 136 (82.4) | 0.001 |
| Transient ischemic attack or stroke, n (%) | 60 (8.6) | 22 (13.3) | 0.064 |
| Cancer, n (%) | 124 (17.8) | 33 (20.0) | 0.514 |
| Osteoporosis, n (%) | 157 (22.6) | 55 (33.3) | 0.004 |
| Asthma, n (%) | 49 (7.0) | 12 (7.3) | 0.917 |
| Chronic Obstructive Pulmonary Disease, n (%) | 67 (9.6) | 17 (10.3) | 0.792 |
| Chronic heart failure, n (%) | 99 (14.2) | 39 (23.6) | 0.003 |
| Coronary artery disease, n (%) | 74 (10.6) | 28 (17.0) | 0.024 |
| Myocardial infarction, n (%) | 54 (7.8) | 18 (10.9) | 0.189 |
| Diabetes, n (%) | 127 (18.2) | 56 (33.9) | <0.001 |
| SPPB | 11.0 (9.0; 12.0) | 9.0 (5.5; 11.0) | <0.001 |
| ASMM (kg) | 18.3 (15.6; 22.2) | 18.6 (15.6; 22.3) | 0.719 |
| Handgrip strength (kg) | 25.0 (20.0; 32.4) | 22.0 (18.0; 28.0) | <0.001 |
| Resistance (Ohms) | 505.3 (454.0; 555.3) | 482.0 (430.0; 539.5) | 0.004 |
| Reactance (Ohms) | 43.0 (37.0; 48.0) | 38.1 (32.0; 45.6) | <0.001 |
| PhA (degrees) | 4.8 (4.4; 5.2) | 4.5 (4.0; 4.9) | <0.001 |
| PhA <= 4.0°, n (%) | 75 (10.8) | 48 (29.1) | <0.001 |
| PhA <= 4.1°, n (%) | 102 (14.7) | 61 (37.0) | <0.001 |
| PhA <= 4.2°, n (%) | 138 (19.8) | 68 (41.2) | <0.001 |
| PhA <= 4.3°, n (%) | 170 (24.4) | 72 (43.6) | <0.001 |
| PhA <= 4.4°, n (%) | 209 (30.0) | 79 (47.9) | <0.001 |
| PhA <= 4.5°, n (%) | 254 (36.5) | 85 (51.5) | <0.001 |
| PhA <= 4.6°, n (%) | 290 (41.7) | 94 (57.0) | <0.001 |
| PhA <= 4.7°, n (%) | 328 (47.1) | 103 (62.4) | <0.001 |

EQ-5D - VAS: Visual Analogue Scale of the EuroQol-5D questionnaire; BMI: body mass index; MNA: Mini Nutritional Assessment; BADL: Basic Activities of Daily Living; IADL: Instrumental Activities of Daily Living; MMSE: Mini Mental State Examination; GDS: Geriatric Depression Scale; SPPB: Short Physical Performance Battery; ASMM: Appendicular Skeletal Muscle Mass; PhA: phase angle.

**Supplementary Table 3. General characteristics of the participants at baseline according to the occurrence of sarcopenia at 24-months follow-up (outcome matched by age and sex).**

| **Variable** | **No sarcopenia at 24-months follow-up** | **Sarcopenia at 24-months follow-up** | **P** |
| --- | --- | --- | --- |
|  | **(n =45)** | **(n =45)** |  |
| Age (years) | 84.0 (78.0; 85.0) | 84.0 (77.5; 85.5) | 0.999 |
| Sex (female), n (%) | 31 (68.9) | 31 (68.9) | 0.999 |
| Education level (years) | 12.0 (9.2; 16.0) | 10.0 (8.0; 14.0) | 0.020 |
| Marital status, n (%) |  |  | 0.950 |
| *Single* | 3 (6.7) | 4 (8.9) |  |
| *Married or cohabiting* | 24 (53.3) | 23 (51.1) |  |
| *Divorced or separated* | 3 (6.7) | 2 (4.4) |  |
| *Widow* | 15 (33.3) | 16 (35.6) |  |
| Economic status, n (%) |  |  | 0.368 |
| *Very good/good/sufficient* | 37 (82.2) | 40 (88.9) |  |
| *Mediocre/bad* | 8 (17.8) | 5 (11.1) |  |
| Living alone, n (%) | 13 (28.9) | 7 (15.6) | 0.143 |
| Quality of Life (EQ-5D - VAS) | 79.8 (65.1; 89.4) | 70.0 (50.0; 85.0) | 0.250 |
| BMI (kg/m^2^) | 27.3 (24.7; 30.1) | 26.2 (24.2; 28.9) | 0.342 |
| MNA at risk of malnutrition, n (%) | 4 (8.9) | 6 (13.3) | 0.479 |
| Dependency in 1 or more BADL, n (%) | 1 (2.2) | 3 (6.7) | 0.306 |
| Dependency in 1 or more IADL, n (%) | 14 (31.1) | 20 (44.4) | 0.167 |
| MMSE <24, n (%) | 2 (4.4) | 5 (11.1) | 0.226 |
| GDS score >5, n (%) | 5 (11.1) | 9 (20.0) | 0.227 |
| Hypertension, n (%) | 32 (71.1) | 33 (73.3) | 0.691 |
| Transient ischemic attack or stroke, n (%) | 4 (8.9) | 3 (6.7) | 0.716 |
| Cancer, n (%) | 8 (17.8) | 7 (15.6) | 0.777 |
| Osteoporosis, n (%) | 10 (22.2) | 16 (35.6) | 0.145 |
| Asthma, n (%) | 3 (6.7) | 2 (4.4) | 0.645 |
| Chronic Obstructive Pulmonary Disease, n (%) | 5 (11.1) | 2 (4.4) | 0.250 |
| Chronic heart failure, n (%) | 7 (15.6) | 3 (6.7) | 0.192 |
| Coronary artery disease, n (%) | 5 (11.1) | 1 (2.2) | 0.091 |
| Myocardial infarction, n (%) | 4 (8.9) | 1 (2.2) | 0.175 |
| Diabetes, n (%) | 8 (17.8) | 9 (20.0) | 0.788 |
| SPPB | 11.0 (9.0; 12.0) | 9.0 (7.0; 11.0) | 0.031 |
| ASMM (kg) | 18.8 (15.9; 22.6) | 15.1 (14.1; 17.9) | 0.048 |
| Handgrip strength (kg) | 25.5 (20.3; 34.0) | 18.0 (16.0; 22.0) | 0.034 |
| Resistance (Ohms) | 501.6 (452.1; 555.6) | 544.0 (505.9; 580.2) | <0.001 |
| Reactance (Ohms) | 43.0 (37.5; 48.6) | 43.5 (37.5; 48.5) | 0.800 |
| PhA (degrees) | 4.8 (4.4; 5.3) | 4.4 (4.0; 5.0) | 0.024 |
| PhA <= 4.0°, n (%) | 4 (8.9) | 12 (26.7) | 0.027 |
| PhA <= 4.1°, n (%) | 6 (13.3) | 15 (33.3) | 0.025 |
| PhA <= 4.2°, n (%) | 8 (17.8) | 17 (37.8) | 0.034 |
| PhA <= 4.3°, n (%) | 11 (23.9) | 20 (44.4) | 0.039 |
| PhA <= 4.4°, n (%) | 13 (28.9) | 24 (53.3) | 0.015 |
| PhA <= 4.5°, n (%) | 16 (35.6) | 26 (57.8) | 0.028 |
| PhA <= 4.6°, n (%) | 18 (40.0) | 27 (60.0) | 0.058 |
| PhA <= 4.7°, n (%) | 21 (46.7) | 29 (64.4) | 0.072 |

EQ-5D - VAS: Visual Analogue Scale of the EuroQol-5D questionnaire; BMI: body mass index; MNA: Mini Nutritional Assessment; BADL: Basic Activities of Daily Living; IADL: Instrumental Activities of Daily Living; MMSE: Mini Mental State Examination; GDS: Geriatric Depression Scale; SPPB: Short Physical Performance Battery; ASMM: Appendicular Skeletal Muscle Mass; PhA: phase angle.

**Supplementary Table 4 Association between phase angle and phase angle cut-offs with occurrence of sarcopenia at 24-months follow-up. Logistic regression models with age, sex, education, MMSE and osteoporosis (outcome matched by age and sex).**

|  | **Occurrence of at least 1 sarcopenia at 24-months follow-up** | | |
| --- | --- | --- | --- |
| **Predictors** | **Model 1**  **OR (95%CI)** | **Model 2**  **OR (95%CI)** | **Model 3**  **OR (95%CI)** |
| PhA, degrees | 0.66 (0.39 – 1.09) | 0.81 (0.51 – 1.30) | 0.67 (0.37 – 1.20) |
| PhA, <= 4.0° | **3.39 (1.04 – 11.1)** | 2.17 (0.62 – 7.61) | 2.67 (0.71 – 10.0) |
| PhA, <= 4.1° | **3.24 (1.13 – 9.31)** | 2.09 (0.67 – 6.46) | 2.60 (0.79 – 8.55) |
| PhA, <= 4.2° | **2.66 (1.02 – 6.94)** | 1.58 (0.55 – 4.53) | 1.89 (0.63 – 5.70) |
| PhA, <= 4.3° | **2.67 (1.08 – 6.61)** | 1.77 (0.66 – 4.74) | 2.10 (0.75 – 5.85) |
| PhA, <= 4.4° | **2.88 (1.21 – 6.87)** | 1.83 (0.70 – 4.76) | 2.21 (0.81 – 6.04) |
| PhA, <= 4.5° | **2.54 (1.09 – 5.93)** | 1.49 (0.57 – 3.87) | 1.94 (0.70 – 5.36) |
| PhA, <= 4.6° | 2.21 (0.96 – 5.13) | 1.31 (0.51 – 3.38) | 1.63 (0.60 – 4.43) |
| PhA, <= 4.7° | 2.13 (0.92 – 4.96) | 1.18 (0.45 – 3.10) | 1.41 (0.51 – 3.88) |

OR: odds ratio; CI: confidence interval; PhA: phase angle

Model 1 – Crude model

Model 2 – Age and sex adjusted model

Model 3 – Model adjusted for age, sex, education, MMSE and osteoporosis

**Supplementary Table 5. Association between phase angle and phase angle cut-offs with occurrence of sarcopenia at 24-months follow-up. Logistic regression models with handgrip, ASMM, and SPPB (outcome matched by age and sex).**

|  | **Occurrence of at least 1 sarcopenia at 24-months follow-up** | | | |
| --- | --- | --- | --- | --- |
| **Predictors** | **Model A***  **OR (95%CI)** | **Model B****  **OR (95%CI)** | **Model C*****  **OR (95%CI)** | **Model D******  **OR (95%CI)** |
| PhA, degrees | 0.81 (0.49 – 1.34) | 0.71 (0.41 – 1.22) | 0.76 (0.46 – 1.24) | 0.87 (0.50 – 1.51) |
| PhA, <= 4.0° | 2.18 (0.60 – 7.91) | **3.67 (1.00 – 13.5)** | 2.69 (0.78 – 9.30) | 2.34 (0.59 – 9.28) |
| PhA, <= 4.1° | 2.06 (0.65 – 6.54) | **3.16 (1.00 – 9.97)** | 2.38 (0.78 – 7.25) | 1.93 (0.56 – 6.64) |
| PhA, <= 4.2° | 1.61 (0.55 – 4.67) | 2.40 (0.85 – 6.79) | 1.82 (0.65 – 5.12) | 1.37 (0.44 – 4.32) |
| PhA, <= 4.3° | 1.69 (0.62 – 4.61) | 2.57 (0.96 – 6.88) | 1.71 (0.63 – 4.60) | 1.36 (0.45 – 4.11) |
| PhA, <= 4.4° | 1.85 (0.71 – 4.84) | **2.86 (1.11 – 7.37)** | 1.91 (0.74 – 4.89) | 1.56 (0.54 – 4.50) |
| PhA, <= 4.5° | 1.71 (0.67 – 4.36) | 2.43 (0.97 – 6.09) | 1.76 (0.71 – 4.39) | 1.43 (0.52 – 3.97) |
| PhA, <= 4.6° | 1.57 (0.62 – 3.99) | 2.11 (0.85 – 5.24) | 1.51 (0.61 – 3.76) | 1.27 (0.46 – 3.49) |
| PhA, <= 4.7° | 1.44 (0.56 – 3.69) | 1.87 (0.75 – 4.65) | 1.44 (0.58 – 3.59) | 1.12 (0.41 – 3.10) |

OR: odds ratio; CI: confidence interval; PhA: phase angle

* adjusted for handgrip

** adjusted for ASMM

*** adjusted for SPPB

**** adjusted for handgrip, ASMM, and SPPB

**Supplementary Table 6. Association between phase angle and phase angle cut-offs with occurrence of sarcopenia at 24-months follow-up. Logistic regression models with handgrip, ASMM, SPPB, age, sex, education, MMSE and osteoporosis (outcome matched by age and sex).**

|  | **Occurrence of at least 1 sarcopenia at 24-months follow-up** | | | |
| --- | --- | --- | --- | --- |
| **Predictors** | **Model 3A***  **OR (95%CI)** | **Model 3B****  **OR (95%CI)** | **Model 3C*****  **OR (95%CI)** | **Model 3D******  **OR (95%CI)** |
| PhA, degrees | 0.88 (0.49 – 1.57) | 0.70 (0.34 – 1.41) | 0.72 (0.41 – 1.28) | 1.18 (0.51 – 2.70) |
| PhA, <= 4.0° | 1.39 (0.33 – 5.87) | 4.62 (0.94 – 22.7) | 2.45 (0.64 – 9.43) | 2.07 (0.35 – 12.2) |
| PhA, <= 4.1° | 1.56 (0.42 – 5.80) | **4.15 (1.00 – 17.1)** | 2.26 (0.66 – 7.67) | 1.98 (0.39 – 10.1) |
| PhA, <= 4.2° | 1.16 (0.33 – 4.04) | 2.69 (0.75 – 9.69) | 1.58 (0.50 – 4.97) | 1.26 (0.27 – 5.79) |
| PhA, <= 4.3° | 1.12 (0.34 – 3.63) | 2.71 (0.83 – 8.83) | 1.64 (0.55 – 4.83) | 0.93 (0.21 – 4.12) |
| PhA, <= 4.4° | 1.16 (0.36 – 3.69) | 2.72 (0.85 – 8.70) | 1.77 (0.62 – 5.08) | 0.96 (0.23 – 4.04) |
| PhA, <= 4.5° | 1.08 (0.34 – 3.48) | 2.08 (0.65 – 6.63) | 1.60 (0.56 – 4.58) | 0.80 (0.19 – 3.43) |
| PhA, <= 4.6° | 0.92 (0.29 – 2.92) | 1.56 (0.50 – 4.86) | 1.33 (0.47 – 3.76) | 0.56 (0.13 – 2.44) |
| PhA, <= 4.7° | 0.84 (0.26 – 2.70) | 1.29 (0.41 – 4.05) | 1.16 (0.41 – 3.30) | 0.57 (0.13 – 2.39) |

OR: odds ratio; CI: confidence interval; PhA: phase angle

* adjusted for handgrip, age, sex, education, MMSE and osteoporosis

** adjusted for adjusted for ASMM, age, sex, education, MMSE and osteoporosis

*** adjusted for SPPB, age, sex, education, MMSE and osteoporosis

**** adjusted for handgrip, ASMM, SPPB, age, sex, education, MMSE and osteoporosis
